# The roles and applications of extracellular vesicles in cancer

**DOI:** 10.1002/1878-0261.70007

**Published:** 2025-02-24

**Authors:** Clotilde Théry, Daniel Louvard

**Affiliations:** ^1^ Institut Curie Research Center PSL Research University, INSERM U932, Immunity and Cancer, and CurieCoreTech Extracellular Vesicles Paris France; ^2^ Institut Curie Research Center PSL Research University, CNRS UMR 144, Cell Biology and Cancer Paris France

**Keywords:** cell communication, exosomes, targeted drug delivery, tumor biomarkers, tumor microenvironment

## Abstract

Extracellular vesicles (EVs) have been studied for several decades and are attracting growing interest among life scientists and oncologists. Understanding the extent of diversity of their cellular origins, structure, molecular composition, and consequently functions is still under progress. EVs offer numerous diagnostic and therapeutic possibilities, but many fundamental questions about their functions need to be resolved in order to effectively and safely implement their applications in the treatment of human diseases.

## Graphical abstract

Extracellular vesicles (EVs) are minute versions of cells limited by a lipid bilayer containing cytoplasm from the cell that releases them, but without a nucleus and thus unable to self‐reproduce. EVs contain multiple molecules (proteins, lipids, glycans, and nucleic acids) they can induce complex responses in cells. Hence, EVs provide new biological tools for diagnosis and therapies of cancers.

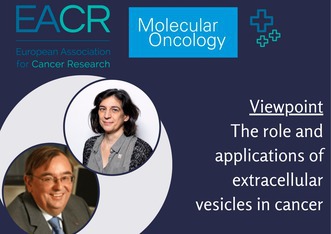

**Abbreviations**
EVs: extracellular vesicles
MHC: Major Histocompatibility Complex

## 1 Definition of extracellular vesicles (EVs)

**Fig. 1:**
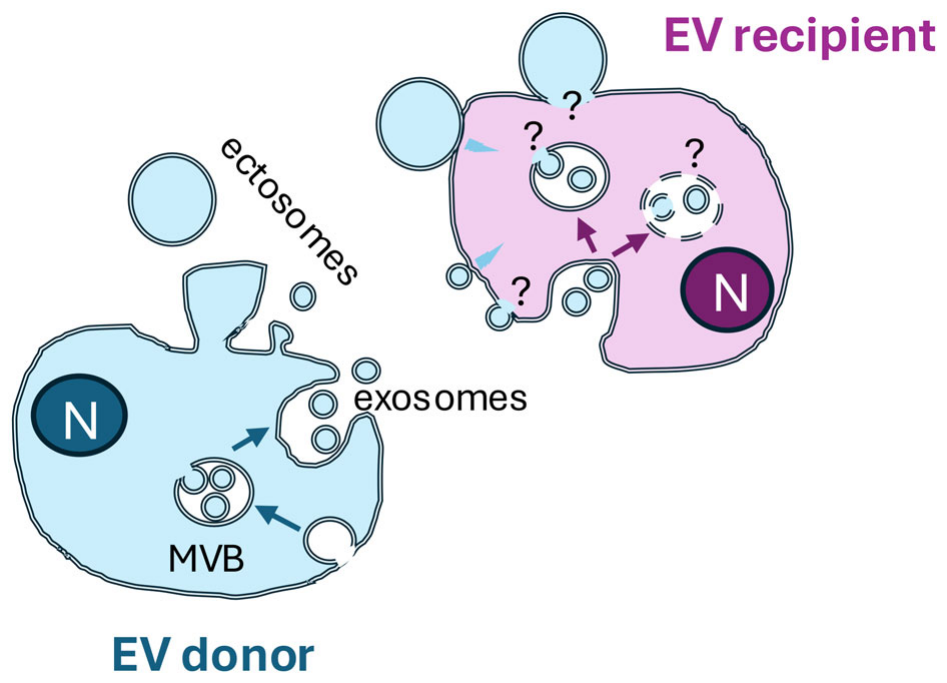
This figure illustrates a cell (EV donor) releasing extracellular vesicles (EVs) from its plasma membrane: ectosomes, and from internal multivesicular bodies (MVB): exosomes. Once outside the producing cell, these EVs can contact another cell (EV recipient), and induce direct signaling from the surface receptors (lightning arrow), or they can be internalized and digested/degraded and/or transfer their content inside the recipient cell. The mechanisms of this transfer are still poorly understood.

In multicellular organisms, communication must occur between cells and between organs, to ensure maintenance of homeostasis, and to allow reaction to pathological situations, for instance, those induced by the development of a tumor. Cells within an organ exchange signals with their neighbors by interactions with their respective surface receptors, or with soluble mediators such as growth factors and cytokines. These signals travel through the tissue or through circulating fluids (e.g., blood and lymph) to reach their cognate receptor on cells in other organs. Extracellular vesicles (EVs) represent another more complex mode of communication combining the properties of both previous examples. EVs are minute versions of cells (their diameter is 10 to 100 times smaller than the cell's diameter), limited by a lipid bilayer and containing cytoplasm from the cell that releases them, but without a nucleus and thus unable to self‐reproduce (Fig. 1). EVs expose and contain multiple molecules (proteins, lipids, glycans, and nucleic acids), which can thus induce complex responses in cells. EVs can form by budding outwards from the plasma membrane of the secreting cell (“ectosomes” in Fig. 1: EV donor cell) or they can form inside intracellular compartments, which then fuse with the plasma membrane, to release outside the internal vesicles (“exosomes” in Fig. 1: EV donor cell). The term EVs (recommended by the International Society for EVs [1]) therefore encompasses a multitude of extracellular particles sharing the lipid‐bilayered structure, but with different subcellular origins, size, molecular content, and therefore different effects on the cells they encounter. Note that, for communication purposes, the term “exosomes” is often used improperly instead of the generic term EVs in many publications, especially from private companies. Recent excellent review articles describe in more details our current knowledge on these features of EVs [2, 3].

## 2 Functions of EVs in cancer

Historically (see Ref. [4] for a detailed historical perspective on EVs), EVs were first described in normal tissues (cartilage and lung) or in blood (as platelets' products), but they were also soon observed produced by cancer cell lines [5]. The current literature suggests that all cells can secrete one or many types of EVs, with variable rates of production by each individual cell, which cannot be predicted based on the cell type or its tumor nature. A range of 1 to 70 in total number of released particles was observed across 50 different tumor cells previously [6]. Indeed, even though many reviews claim that tumor cells release more EVs than normal cells, this statement is not demonstrated, and in fact primary cells such as immune dendritic cells release, in our hands, many more EVs of many different types than any tumor cell line we have tested so far (personal observation, unpublished data). Nonetheless, the production of EVs by tumor cell lines, explants, tumors *in vivo*, as well as their effects on other cells *in vitro* or *in vivo* have been extensively analyzed (see recent review [7]). In most studies, tumor‐derived EVs affect neighboring cells of tumor or nontumor origin in a manner that promotes local tumor progression. Tumor‐derived EVs also participate in seeding of the metastatic niche in other organs. A few studies, however, have described immune‐mediated antitumor effects of tumor‐derived EVs [8, 9, 10]. In addition, it is not only tumor cells that release EVs but also the other cells in the tumor tissue (fibroblasts, endothelial cells, and immune cells). Tumor fibroblast‐derived EVs are mostly described as promoting tumor progression [11], whereas immune cell‐derived EVs could have both pro‐ and antitumoral effects [12]. The outcome of these numerous different EVs of different cellular and subcellular origins, with possible opposite functions, likely present simultaneously in a tumor microenvironment that changes during tumor evolution, thus remains to be studied using a wide variety of models. A strong technical difficulty resides in following small and heterogeneous structures such as EVs *in vivo* inside their tissue of origin during and after traveling to other organs. The awareness of investigators to these issues will likely lead to major developments and expected advances in the coming years [13].

## 3 EVs as source of circulating biomarkers

In parallel with studies on their functions, since tumor‐derived (or tumor‐induced) EVs can leave the tissue and reach biofluids, they are also extensively explored as novel liquid biopsy biomarkers of the tumor evolution and/or its response to treatment [14]. However, there is still a long way to go before establishing the appropriate EV isolation methods, which can be used routinely in the hospital context, while providing EV‐specific and reproducible information [15]. A test quantifying mRNA/DNA cargo of urine EVs for early diagnosis of prostate cancer patients has been approved and is nowadays used in the United States [14]. As protein cargo of EVs, in the booming era of antitumor immunotherapy, ongoing studies may eventually provide important information on the value of checkpoint inhibitor molecules (especially PD‐L1) circulating freely or associated with EVs in blood, as biomarker or potential inhibitors of response to immunotherapy. Indeed, circulating PD‐L1 could act as a decoy for anti‐PD‐L1 or counteract anti‐PD‐1 antibodies used for treatment [16].

## 4 EVs as therapeutic tools

Lastly, an important feature of EVs in cancer is their potential as therapeutic tools. The very first clinical trials using EVs were published in 2005 and 2016, with the aim to reinitiate immune responses against the tumor by injecting EVs from the patient's own dendritic cells loaded with antigens from its own tumor [17]. In this setting, MHC–peptide complexes exposed on the surface of EVs were meant to activate a cognate receptor on the patient's tumor‐specific T lymphocytes to boost their killing activity against the tumor. These studies were the first convincingly showing the feasibility of preparing and safety of injecting EVs in patients. These features of EV‐based therapies (feasibility and safety) were recently confirmed by a wider meta‐analysis of clinical trials [18]. However, the clinical outcomes of the dendritic cell EV trial were not as convincing as those obtained by the simultaneously developed anticheckpoint immunotherapies, and the trial was halted after a few dozen patients. The subsequent knowledge on the heterogeneity of EVs and the possible stronger effect of large EVs, rather than the small exosomes used in the trial, could now point to novel options for implementation. Another way EVs are used as therapies is for their ability to deliver their content into target cells, even though the actual mechanisms of this delivery are still unknown (Fig. 1: EV recipient cell). Indeed, many biotech companies aiming to develop EV‐based therapies have been created worldwide in the last decade. Despite the financing difficulties these biotechs may face (one of the most promising American ones went bankrupt and closed down its clinical trial in 2023 for lack of investors), we should keep hope that some of them will eventually succeed in developing novel therapies. Two clinical trials are currently ongoing, one to deliver curcumin loaded into plant‐EVs to colon cancer patients (NCT01294072), and another one to deliver anti‐KRAS siRNA into pancreatic cancer cells via mesenchymal stem cell‐derived EVs (NCT03608631). Many small human therapeutic trials are also published; however, only about 5% of them deal with cancer [19]. As stated by Fusco et al. [19] “[…] those 40 studies […] are all small pilot trials with a large heterogeneity in terms of administration route and target disease […and] absence of a placebo control in most of the studies.” Hopefully, EV biotechs will benefit from experience gained by other companies developing therapeutic uses of liposomes, which are an artificial/synthetic equivalent of EVs, especially on the marketing and development paths to follow. One striking example of a very successful development is the liposome‐encapsulated RNA vaccine, which was very swiftly developed during COVID‐19 thanks to decades of research by these companies on liposome/RNA‐based antitumor vaccines.

## 5 Conclusion

The legitimate hopes to use EVs for diagnostic and/or therapeutic purposes imply a strong need to further study the mode of action and thus the basic biology to unravel the properties of EVs. Of particular importance one may mention to analyze EVs either produced by native unmanipulated cells, or by engineered cells with the goal to produce EVs with a given cargo, as currently aimed by several laboratories worldwide [20]. It is interesting to note that academic researchers are often key players in early‐stage biotech companies, illustrating the dynamism of the field and its potential for innovation.

## Conflict of interest

CT is inventor on two filed patents on the therapeutic use of EVs.

## Author contributions

CT: prepared the first draft of text and figure, finalized the submitted version. DL: discussed, contributed, and corrected the successive versions.
